# Effects of Early and Late Labor Epidural Analgesia on Multiparous Women: A Retrospective Monocentric Study

**DOI:** 10.7759/cureus.84825

**Published:** 2025-05-26

**Authors:** Eswary Varma, Zeenat Fatima, Nilanjana Singh

**Affiliations:** 1 Department of Obstetrics and Gynecology, Kanad Hospital, Al Ain, ARE

**Keywords:** analgesia counseling in multipara in labor, bupivacaine labor epidural effects, early versus late labor epidural, need for intervention with epidural timings, timing of epidural and outcomes

## Abstract

Background

Epidural analgesia is a popular and effective method of pain relief in labor, but the optimal timing of its administration remains unclear for multiparous women. Some evidence suggests that initiating epidurals very early in labor may be associated with increased interventions. This study aimed to evaluate whether early labor epidural analgesia (initiated before active labor) affects delivery outcomes in multiparous women compared to late epidural or no epidural analgesia.

Methodology

We conducted a retrospective, single-center, cohort study of term multiparous women (at least 37 weeks of gestation) with singleton, cephalic pregnancies and no prior cesarean delivery, who gave birth between November 1, 2023, and April 30, 2024. Participants were divided into the following three groups based on use of neuraxial analgesia: no neuraxial analgesia (n = 421), early neuraxial analgesia (less than 3 cm of cervical dilation; n = 102), or late neuraxial analgesia (3 cm of dilation or more; n = 145). Primary outcomes were mode of delivery (vaginal versus cesarean), use of oxytocin for labor augmentation, and postpartum hemorrhage of at least 1,000 mL. Secondary outcomes included admission to a neonatal intensive care unit, Apgar scores below 7 at five minutes, and meconium-stained amniotic fluid.

Results

A total of 668 multiparous women were included. The early neuraxial analgesia group had the highest rates of labor induction (46/102, 45.10%), labor augmentation (41/102, 40.20%), and cesarean delivery (10/102, 9.80%), while the no neuraxial analgesia group had the lowest rates (98/421, 23.28%; 60/421, 14.25%; and 12/421, 2.85%, respectively). Spontaneous vaginal delivery rates were 88/102 (86.27%) for early neuraxial analgesia, 138/145 (95.17%) for late neuraxial analgesia, and 405/421 (96.20%) for no neuraxial analgesia. Postpartum hemorrhage of at least 1,000 mL occurred in 6/102 (5.88%) of the early neuraxial analgesia group, 6/145 (4.14%) of the late neuraxial analgesia group, and 8/421 (1.90%) of those without neuraxial analgesia. Neonatal intensive care unit admissions were slightly higher in the early neuraxial analgesia group (4/102, 3.92%) compared with late neuraxial analgesia (3/145, 2.07%) and no neuraxial analgesia (3/421, 0.71%) groups. Five-minute Apgar scores below 7 remained low in all groups, ranging from 0/102 (0.00%) to 1/145 (0.69%).

Conclusions

Among multiparous women, initiating epidural analgesia in early labor was associated with higher rates of labor augmentation and operative delivery, whereas late epidural analgesia or no epidural analgesia was linked to fewer interventions. Despite these differences, serious maternal complications and neonatal outcomes remained favorable across all groups. These findings suggest that delaying epidural initiation until active labor may help minimize interventions without compromising maternal or neonatal safety.

## Introduction

Epidural analgesia is the gold standard for pain relief during labor, with widespread use in modern obstetrics [1]. Its application not only improves maternal comfort but also plays a key role in overall perinatal care. However, the optimal timing of epidural initiation, whether early in the latent phase or later during active labor, remains a debated clinical issue.

Early concerns suggested that initiating an epidural too soon might prolong labor and increase operative delivery rates [2]. Subsequent randomized trials and meta-analyses have challenged these notions, showing that early neuraxial analgesia does not necessarily elevate cesarean or instrumental delivery risks [3]. Notably, a comprehensive Cochrane review [3], which included 15,752 women across nine randomized trials, found no increased risk of cesarean delivery with early epidural analgesia (risk ratio (RR) = 1.02, 95% confidence interval (CI) = 0.96-1.08), and no significant difference in instrumental delivery rates (RR = 0.93, 95% CI = 0.86-1.01). This evolving evidence has reshaped clinical practices in labor management.

Beyond delivery mode and duration, the timing of epidural analgesia may affect other maternal and neonatal outcomes. Maternal fever, potentially linked to prolonged epidural exposure, can lead to neonatal sepsis evaluations and neonatal intensive care unit (NICU) admissions [4]. Although studies generally report stable Apgar scores across timing groups, subtle differences in postpartum hemorrhage and neonatal observations have been noted.

Most research has focused on nulliparous women, leaving a gap in understanding for multiparous populations. Multiparous women typically experience faster, more efficient labors, yet may respond differently to the timing of analgesia. A recent analysis in this group showed no significant difference in labor duration between early and late epidural initiation [4], underscoring the need for focused investigation. Moreover, a recent retrospective study by Cheng et al. [5] in 1,119 multiparous women found that while neuraxial analgesia generally prolonged the first and second stages of labor relative to no analgesia, early versus late initiation of epidural analgesia did not lead to higher rates of intrapartum cesarean delivery, intrapartum fever, or adverse neonatal outcomes. Such findings further reinforce the notion that early epidural use may be safe for multiparous women, but additional data are needed to clarify potential differences in labor interventions and postpartum outcomes.

To address this gap, our study retrospectively examined multiparous women with full-term, singleton, cephalic pregnancies admitted between November 1, 2023, and April 30, 2024, in a tertiary care hospital in Al Ain, UAE. Because multiparous women generally experience different labor dynamics compared to nulliparous women, often with more rapid cervical dilation and potentially differing responses to analgesia, they represent an important yet under-investigated population. We compared outcomes among women receiving early epidural analgesia (initiated before 3 cm dilation), late epidural analgesia (after 3 cm dilation), and those not receiving any epidural analgesia.

Our primary focus was to assess whether early initiation affects labor interventions (cesarean delivery, instrumental delivery, labor augmentation) and neonatal morbidity indicators (NICU admission, Apgar scores) compared to late initiation or no epidural analgesia. We hypothesized that early initiation would be non-inferior to later initiation regarding obstetric outcomes, aiming to provide evidence-based guidance for timing epidural administration in this specific population.

## Materials and methods

Study design and setting

This was a retrospective, single-center, observational study conducted at a maternity hospital. We reviewed all eligible labor and delivery records from November 1, 2023, through April 30, 2024, corresponding to the study period. The analysis was confined to this single institution (monocentric design).

Participants and inclusion criteria

All multiparous women who delivered during the study period were considered for inclusion. Inclusion criteria required a singleton pregnancy in cephalic presentation at term gestation (37+0 to 41+6 weeks), resulting in a live-born infant. Women were excluded if they were primiparous, had a preterm delivery (gestational age <37 weeks), a multiple pregnancy, a stillbirth, or any previous cesarean delivery. All women meeting these criteria within the study timeframe were included, with no further exclusions. Cervical dilation was typically assessed by a midwife or the attending obstetrician to maintain standardization.

Epidural analgesia protocol

Epidural labor analgesia was available on maternal request and was administered by the on-duty anesthesiologist according to the hospital’s standard protocol. At our institution, we initiate epidural analgesia by first administering 500 mL of Ringer’s lactate solution, then placing the catheter at the L3-4 or L4-5 interspace. An initial bolus dose of 20 mg bupivacaine plus 100 µg fentanyl is administered by the anesthesiologist. Subsequently, a continuous infusion of bupivacaine 0.1% with fentanyl 2 µg/mL is maintained at 10 mL/hour, with patients able to self-administer an additional 6 mL bolus (lockout interval of 20 minutes) as needed for breakthrough pain. A standard Visual Analog Scale was used to assess pain at baseline (upon admission) and periodically during labor, including each time a bolus was requested. The infusion is stopped after any necessary suturing, and the catheter is typically removed one to two hours postpartum.

For the purpose of analysis, we stratified the cohort based on the timing of epidural administration. Early epidural analgesia was defined as epidural initiation in early labor, before 3 cm of cervical dilation, while late epidural analgesia was defined as epidural initiation at ≥3 cm of cervical dilation (active labor). Women who did not receive any epidural analgesia during labor were analyzed as a third comparison group (the no epidural group). Apart from timing (or absence) of epidural placement, all other obstetric management followed routine care protocols.

Data collection

Data were retrospectively extracted from the hospital’s electronic medical record system for all included cases. We collected maternal demographic details (including age and parity) and relevant obstetric data. Labor characteristics such as onset of labor (spontaneous or induced), cervical dilation at the time of epidural request/placement, and the duration of labor (length of first and second stages) were recorded. All data points were collected using a consistent abstraction form to ensure uniformity.

Outcome measures

Key maternal and neonatal outcomes were predefined and assessed for each group. The primary outcomes of interest included the mode of delivery, in particular, the rate of cesarean delivery, and the incidence of postpartum hemorrhage ≥1,000 mL. We also evaluated maternal intrapartum fever (temperature elevation during labor), the presence of meconium-stained amniotic fluid, and several neonatal outcomes. Neonatal assessments included five-minute Apgar scores (with a focus on Apgar <7 at five minutes) and the rate of NICU admissions. Additionally, we compared the duration of labor stages among the groups to determine if epidural timing affected the length of the first or second stage of labor. For missing data points, we conducted a listwise deletion when the missing variable was critical to a specific analysis, ensuring that the final reported results were based on complete records. All outcome measures were defined before data analysis and were obtained directly from the delivery and postpartum records.

Statistical analysis

We performed a descriptive comparison of the three groups, i.e., early epidural (initiation before 3 cm of cervical dilation), late epidural (initiation at 3 cm or more), and no epidural, to identify potential differences in maternal demographics, labor course, and outcomes. Data were derived from the final set of 668 multiparous women, including 102 who received early epidural analgesia, 145 who received late epidural analgesia, and 421 who did not receive epidural analgesia.

Continuous variables, such as maternal age and duration of each labor stage, were summarized using measures of central tendency (mean or median) and dispersion (standard deviation or interquartile range). Categorical variables, such as mode of delivery, postpartum hemorrhage, meconium-stained amniotic fluid, maternal fever, NICU admission, and Apgar scores below 7 at five minutes, were reported as counts and percentages in each of the three groups. All analyses were performed using a standard statistical software package (SPSS Statistics; IBM Corp., Armonk, NY, USA), and the patterns observed were interpreted in light of relevant clinical considerations.

Participant flow

**Figure 1:**
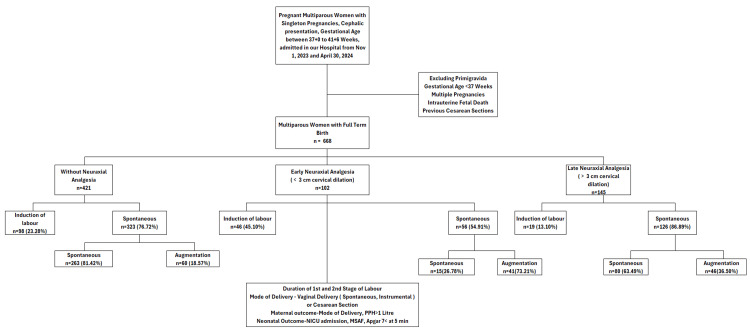
Flow diagram summarizing the inclusion, exclusion, and group assignment of multiparous women who met the study criteria. PPH = postpartum hemorrhage; NICU = neonatal intensive care unit; MASF = meconium-stained amniotic fluid

Figure 1 presents a flow diagram of the study population. Beginning with all multiparous women admitted between November 1, 2023, and April 30, 2024 (n = 668), we excluded those who did not meet the inclusion criteria (gestational age <37 weeks, multiple pregnancies, prior cesarean, or intrauterine fetal demise). Eligible participants (n = 668) were then divided into three groups based on whether they received no neuraxial analgesia (n = 421), early epidural analgesia (initiated before 3 cm of cervical dilation; n = 102), or late epidural analgesia (initiated at 3 cm or more; n = 145). The figure also summarizes the proportion of induced, augmented, and spontaneous labors in each group, illustrating the final analytic sample.

## Results

Participant characteristics

A total of 668 multiparous women delivered during the study period and met the inclusion criteria, of whom 102 were in the early neuraxial analgesia group, 145 in the late neuraxial analgesia group, and 421 in the no neuraxial analgesia group. Baseline maternal age and parity distribution differed slightly across groups but were generally comparable. Table 1 provides a comprehensive overview of maternal and neonatal outcomes according to the timing of neuraxial analgesia. The data encompass mode of delivery, labor characteristics, and selected complications.

**Table 1:** Maternal and neonatal outcomes by the timing of NA. Note: The “Augmented labor” row shows the number and percent only among participants who began labor spontaneously in each group. NA = neuraxial analgesia; NICU = neonatal intensive care unit

Outcome	Early NA (n = 102), n (%)	Late NA (n = 145), n (%)	No NA (n = 421), n (%)
Cesarean delivery	10 (9.80%)	5 (3.45%)	12 (2.85%)
Instrumental delivery	4 (3.92%)	2 (1.38%)	4 (0.95%)
Spontaneous vaginal delivery	88 (86.27%)	138 (95.17%)	405 (96.20%)
Induced labor	46 (45.10%)	19 (13.10%)	98 (23.28%)
Augmented labor	41 (73.21%)	46 (36.50%)	60 (18.57%)
Spontaneous labor	56 (54.91%)	126 (86.89%)	323 (76.72%)
Postpartum hemorrhage ≥1,000 mL	6 (5.88%)	6 (4.14%)	8 (1.90%)
Maternal fever	0 (0.00%)	0 (0.00%)	1 (0.24%)
Apgar <7 at five minutes	0 (0.00%)	1 (0.69%)	1 (0.24%)
NICU admission	4 (3.92%)	3 (2.07%)	3 (0.71%)
Meconium-stained amniotic fluid	6 (5.88%)	18 (12.41%)	17 (4.04%)
First-stage labor	6 hours, 52 minutes	5 hours, 29 minutes	5 hours, 20 minutes
Second-stage labor	19 minutes	24 minutes	12 minutes

Labor characteristics

Induction of labor was most frequent in the early neuraxial analgesia group (46/102, 45.10%), followed by the no neuraxial analgesia (98/421, 23.28%) and late neuraxial analgesia (19/145, 13.10%) groups. Augmentation with oxytocin occurred in 41/102 (40.20%) of early neuraxial analgesia, 46/145 (31.72%) of late neuraxial analgesia, and 60/421 (14.25%) of no neuraxial analgesia participants. Spontaneous labor (defined as labor that began without induction) occurred in 56/102 (54.90%) of early neuraxial analgesia, 126/145 (86.89%) of late neuraxial analgesia, and 323/421 (76.72%) of no neuraxial analgesia participants. Among those who began labor spontaneously, 41/56 (73.21%) in the early neuraxial analgesia group eventually required oxytocin augmentation, compared to 46/126 (36.50%) in the late neuraxial analgesia, and 60/323 (18.57%) in no neuraxial analgesia groups.

Mode of delivery and labor interventions

**Figure 2:**
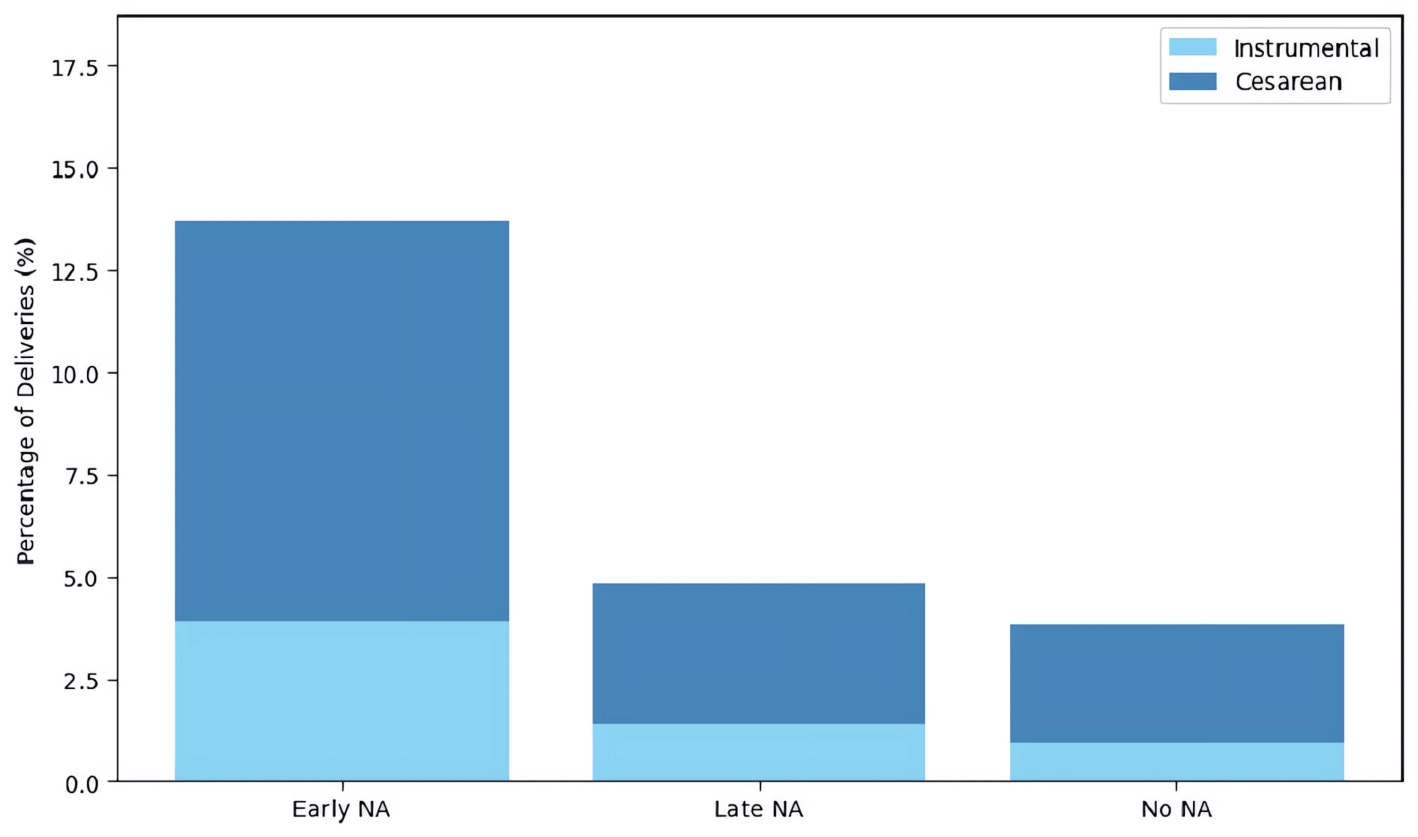
Cesarean and instrumental delivery rates by NA group. NA = neuraxial analgesia

As shown in Table 1, the cesarean delivery rate was 10/102 (9.80%) in the early neuraxial analgesia, 5/145 (3.45%) in late neuraxial analgesia, and 12/421 (2.85%) in no neuraxial analgesia groups. Instrumental delivery occurred in 4/102 (3.92%), 2/145 (1.38%), and 4/421 (0.95%) women, respectively, while spontaneous vaginal birth remained the predominant mode across groups (≥86%). Figure 2 presents these operative deliveries (cesarean + instrumental) as stacked bars.

The chi‑square analysis (Table 2) confirmed that early neuraxial analgesia was associated with the highest incidence of cesarean delivery (chi‑square = 10.40, p = 0.006) and labor augmentation (chi‑square = 42.39, p < 0.000001) compared with late neuraxial analgesia and no neuraxial analgesia. Although instrumental delivery was numerically greater in the early neuraxial analgesia group, the difference did not reach statistical significance (chi‑square = 4.93, p = 0.085).

**Table 2:** Mode of delivery and labor augmentation by the timing of neuraxial analgesia (N = 668). In each cell, “event vs no‑event” shows how many participants experienced the specified outcome (event) versus how many did not (no‑event). The “Augmented labor” row refers to oxytocin administration among all participants in each group, including those whose labor was induced and those who started spontaneously. P-values <0.05 are considered significant; significant p-values are marked with *. ENA = early neuraxial analgesia; LNA = late neuraxial analgesia; NNA = no neuraxial analgesia.

Outcome	ENA (n = 102)	LNA (n = 145)	NNA (n = 421)	Test statistic (χ², df = 2)	P-value
Cesarean delivery	10 vs. 92	5 vs. 140	12 vs. 409	10.40	0.0055*
Instrumental delivery	4 vs. 98	2 vs. 143	4 vs. 417	4.93	0.085
Augmented labor	41 vs. 61	46 vs. 99	60 vs. 361	42.39	<0.00001*

Duration of labor

The average first stage of labor was the longest in the early neuraxial analgesia group at 6 hours, 52 minutes, compared with 5 hours, 29 minutes in the late neuraxial analgesia group and 5 hours, 20 minutes in the no neuraxial analgesia group. The second stage was 19 minutes in early neuraxial analgesia, 24 minutes in late neuraxial analgesia, and 12 minutes in no neuraxial analgesia.

Maternal outcomes

Postpartum hemorrhage (≥1,000 mL) occurred in 6/102 (5.88%) of early neuraxial analgesia, 6/145 (4.14%) of late neuraxial analgesia, and 8/421 (1.90%) of no neuraxial analgesia participants. Maternal fever was not reported in the early neuraxial analgesia or late neuraxial analgesia groups, while 1/421 (0.24%) of the no neuraxial analgesia group experienced fever.

Neonatal outcomes

Apgar scores <7 at five minutes were recorded in 0/102 (0.00%) of early neuraxial analgesia, 1/145 (0.69%) of late neuraxial analgesia, and 1/421 (0.24%) of no neuraxial analgesia infants. NICU admissions occurred in 4/102 (3.92%) of early neuraxial analgesia, 3/145 (2.07%) of late neuraxial analgesia, and 3/421 (0.71%) of no neuraxial analgesia. Meconium-stained amniotic fluid was noted in 6/102 (5.88%) of early neuraxial analgesia, 18/145 (12.41%) of late neuraxial analgesia, and 17/421 (4.04%) of no neuraxial analgesia deliveries.

Impact of age

**Figure 3:**
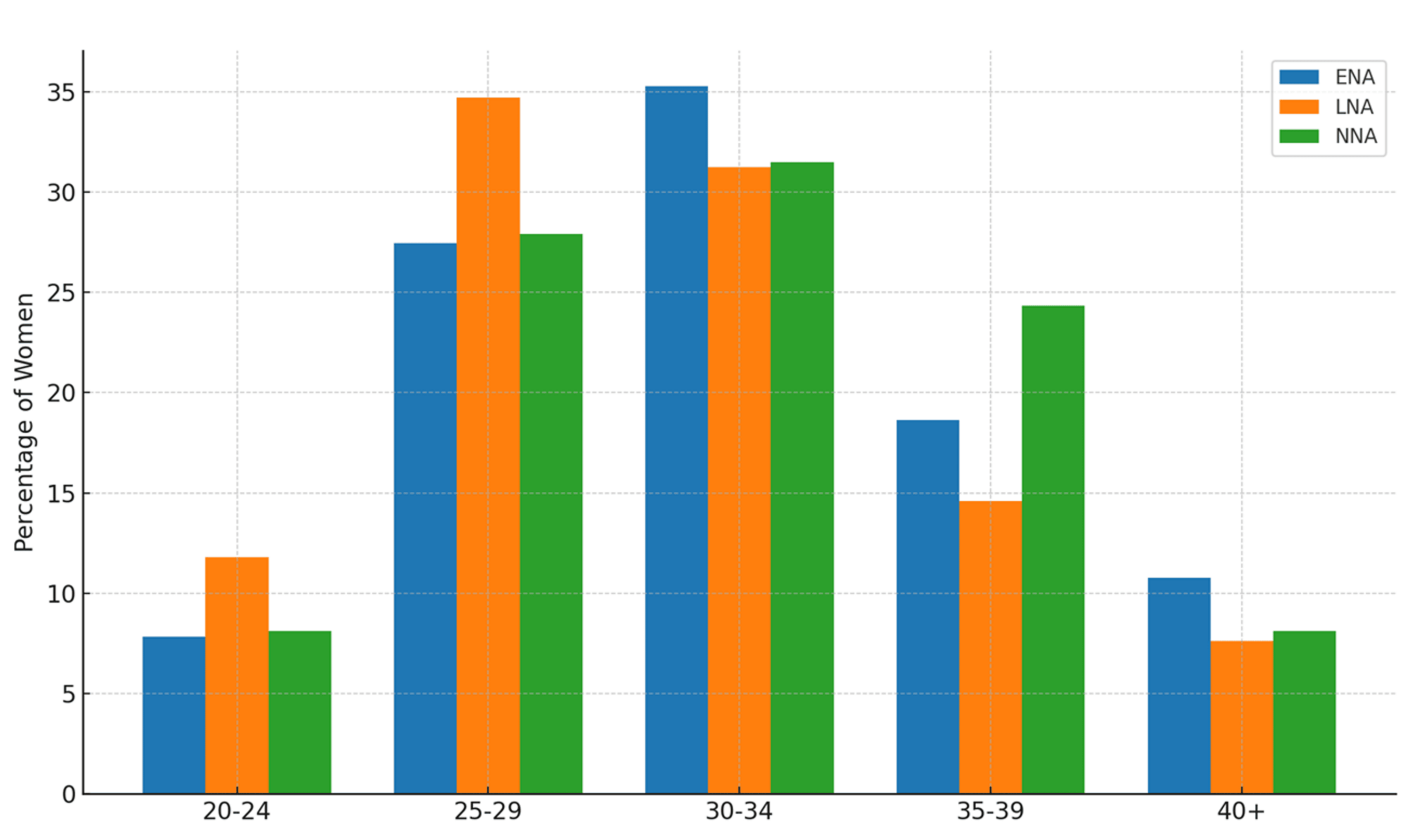
Age distribution by analgesia group. ENA = early neuraxial analgesia; LNA = late neuraxial analgesia; NNA = no neuraxial analgesia

Figure 3 shows that most women in all three groups were in the 25‑34‑year age bracket; however, the early neuraxial analgesia cohort had a slightly larger proportion of women ≥30 years, whereas the no neuraxial analgesia group contained the highest share of women ≥35 years.

Parity

**Figure 4:**
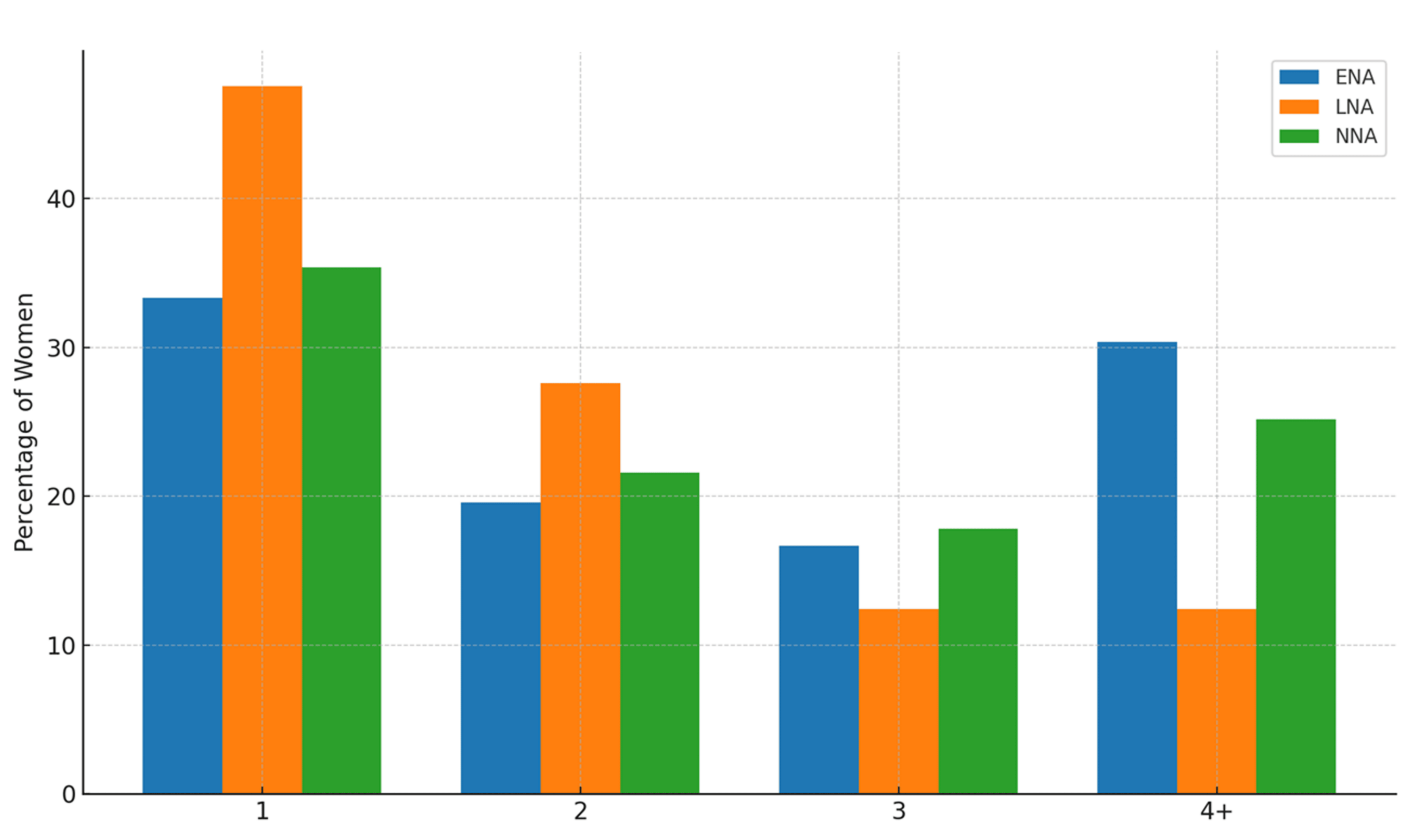
Parity distribution by analgesia group. ENA = early neuraxial analgesia; LNA = late neuraxial analgesia; NNA = no neuraxial analgesia

Figure 4 illustrates a clear parity gradient: late neuraxial analgesia was dominated by low‑parity women (para 1-2), early neuraxial analgesia included proportionally more grand‑multiparas (para ≥4), and no neuraxial analgesia had a balanced distribution but still more high‑parity cases than late neuraxial analgesia.

Overall, these updated data demonstrate higher rates of induction and augmentation among the early neuraxial analgesia cohort, along with a greater proportion of cesarean deliveries compared to late neuraxial analgesia and no neuraxial analgesia. Despite these differences, major maternal complications remained infrequent, and neonatal outcomes were generally favorable across all three groups.

## Discussion

Our retrospective analysis among multiparous women indicates that the timing of epidural analgesia during labor can influence delivery outcomes. Specifically, initiating epidural analgesia early (before approximately 3 cm cervical dilation) was associated with a higher incidence of cesarean delivery (10/102, 9.80% vs. 5/145, 3.45% vs. 12/421, 2.85% among early, late, and no epidural groups, respectively) and a small, non-significant increase in postpartum hemorrhage (6/102, 5.88% vs. 6/145, 4.14% vs. 8/421, 1.90%, respectively) compared with late initiation or no epidural use. These findings appear consistent with concerns raised in some observational research indicating that epidural analgesia may be a risk factor for operative delivery under certain circumstances [2]. By contrast, other evidence, including a Cochrane review and several randomized trials, has reported no significant increase in cesarean or instrumental delivery with early epidural administration [3]. Such differing results may reflect variations in study design (observational vs. randomized), population characteristics (e.g., multiparous vs. nulliparous), definitions of “early” epidural placement, or other institutional practices that influence labor management.

Neonatal outcomes, including five‐minute Apgar scores and NICU admissions, did not differ significantly and remained broadly favorable (e.g., five-minute Apgar scores below 7 were 0/102, 0.00%; 1/145, 0.69%; and 1/421, 0.24%, while NICU admissions were 4/102, 3.92%; 3/145, 2.07%; and 3/421, 0.71%, respectively), suggesting that the differences lie predominantly in maternal and labor management parameters [3].

These findings align with previous studies that have demonstrated the safety of epidural analgesia in labor when modern low‐dose regimens are used [3]. However, our results also indicate that early initiation in multiparous women, who typically have more efficient labors, might predispose them to a higher rate of oxytocin augmentation among those starting labor spontaneously and additional clinical interventions. Similarly, another retrospective cohort study reported that labor analgesia performed at cervical dilations ≤4 cm or ≥9 cm was associated with increased risk of operative delivery and adverse neonatal outcomes [6]. For example, early epidural use was linked to a higher rate of operative deliveries, a pattern that supports the hypothesis that analgesia in the latent phase may blunt the natural augmentation of labor [7]. Such increased reliance on labor augmentation in the early epidural analgesia group underscores the potential trade-off between early pain relief and the need for more active management.

Our study further observed that while spontaneous vaginal delivery remained the predominant mode of birth, the early epidural group experienced a modest increase in augmentation and operative interventions. These observations may be explained by the impact of early analgesia on the endogenous oxytocin surge and uterine contractility. In multiparous women, the rapid progression of labor is a key feature, and early intervention might disrupt this process, necessitating additional clinical management such as oxytocin augmentation or instrumental delivery [5]. Moreover, differences in our hospital’s use of bupivacaine for epidural analgesia (as opposed to ropivacaine used in many other institutions) may partially explain discrepancies in labor or delivery outcomes compared to other studies. A recent meta-analysis by Guo et al. found that while both bupivacaine plus fentanyl and ropivacaine plus fentanyl provide effective analgesia, bupivacaine is associated with a slightly higher degree of motor blockade, potentially contributing to differences in labor progression or operative delivery rates [8]. Ropivacaine is associated with less motor blockade and may alter the progression of labor differently than bupivacaine. Further comparative research is needed to determine whether the choice of local anesthetic influences the rate of required interventions such as oxytocin augmentation or operative deliveries in multiparous women.

In addition, the marginally higher incidence of postpartum hemorrhage in the early epidural group could be related to a prolonged labor course or an increased need for labor augmentation, factors that may affect uterine contractility postpartum [9]. Although this trend did not reach statistical significance, a pattern emerged favoring higher postpartum hemorrhage (5.88% vs. 4.14% vs. 1.90%) and a slightly increased instrumental delivery rate (3.92% vs. 1.38% vs. 0.95%) in the early epidural group compared to late or no epidural use. This warrants further consideration, as similar trends have been observed in other retrospective analyses of epidural timing, underscoring the need for larger prospective trials to clarify whether these findings are clinically meaningful. Nonetheless, this finding warrants cautious interpretation when counseling multiparous women about the timing of analgesia.

From a clinical perspective, these results emphasize the importance of individualized decision-making regarding epidural timing in multiparous patients. While epidural analgesia remains an essential and safe option for intrapartum pain relief, careful consideration of the timing may optimize maternal outcomes by balancing effective pain control with the minimization of labor interventions. Providers should discuss these nuances with patients, ensuring that their preferences are met without compromising labor progress. Notably, neonatal outcomes did not differ significantly between groups, underscoring the reassuring safety profile of epidural analgesia in this population.

Nevertheless, other systematic reviews and meta-analyses also report no significant increase in cesarean or instrumental delivery risk with early epidural initiation. For example, Wassen et al. concluded that early versus late epidural placement had no significant effect on cesarean or operative vaginal delivery rates [10]. Specifically, in a pooled analysis of six studies (five randomized controlled studies and one retrospective cohort) involving 15,399 nulliparous women, Wassen et al. found an RR of 1.02 (95% CI = 0.96-1.08) for cesarean delivery and 0.96 (95% CI = 0.89-1.05) for instrumental vaginal delivery when comparing epidural initiation at ≤3 cm versus ≥4 cm dilation. Their sample sizes ranged from as few as 60 participants to as many as 12,793, allowing for a relatively broad assessment of outcomes [10]. These central tendency measures and their 95% CIs underscore the consistency and reliability of prior findings that early epidural placement does not significantly increase operative delivery rates. Taken together, these findings suggest that early epidural initiation is not universally associated with higher intervention rates, underscoring the complexity of comparing different study populations and analgesic protocols.

Additionally, a 2018 Cochrane review supports that epidural analgesia remains the most effective method of labor analgesia [11], emphasizing its superior pain relief and high maternal satisfaction compared to alternative methods. The American College of Obstetricians and Gynecologists similarly states that neuraxial analgesia may be offered at any time in labor [12], thus reinforcing flexible clinical practice and respect for patient autonomy. Meanwhile, Silva and Halpern emphasize that low-dose local anesthetic-opioid solutions can preserve maternal expulsive efforts and limit motor blockade [13]. Collectively, these findings underscore the importance of individualized decision-making and a patient-centered approach when determining epidural timing and technique.

Limitations

Several limitations warrant consideration. Because this was a retrospective, observational study conducted at a single tertiary center, the findings establish associations rather than causation and may not be fully generalizable. Prospective randomized trials are needed to determine whether the timing of epidural initiation directly influences labor outcomes. Although baseline characteristics were comparable and data collection was rigorous, unmeasured confounders, such as individual pain tolerance, subtle variations in clinical decision-making, and the inherent imprecision of estimated blood loss used to identify postpartum hemorrhage, could still have affected the results. Finally, the small absolute number of adverse events limits the precision of the effect estimates and necessitates cautious interpretation.

## Conclusions

In summary, our study suggests that in multiparous women, early epidural analgesia (administered before active labor is well established) is associated with a modest increase in operative interventions and postpartum hemorrhage compared with later epidural administration or no epidural. Despite these differences, key neonatal outcomes remain unaffected. Clinicians should consider these findings when discussing epidural timing with multiparous patients to ensure that pain relief is balanced with the potential for increased labor interventions. These results support a tailored approach to labor analgesia in multiparous women, emphasizing the importance of patient preference while acknowledging the potential benefits of initiating epidural analgesia during active labor to optimize overall outcomes.
